# Nutritional intake and growth in children with intestinal failure: An exploratory cross-sectional study

**DOI:** 10.1016/j.intf.2026.100363

**Published:** 2026-02-17

**Authors:** Naoko Inamura, Miyuki Takeda, Manami Sasaki, Yumi Nishikawa, Chihiro Tanaka, Mikiko Fuda, Tsuyoshi Sakurai, Ryo Ando, Megumi Nakamura, Hironori Kudo, Ryoichi Nagatomi, Motoshi Wada

**Affiliations:** a Department of Pediatric Surgery, Tohoku University Graduate School of Medicine, Japan; b Shokei Gakuin University, Japan; c Nutrition Management Office, Tohoku University Hospital, Japan; d Department of Pediatric Surgery, Tohoku University School of Medicine, Japan; e Department of Pediatric Surgery, Tohoku University Hospital, Japan; f Tohoku University Promotion Office of Strategic Innovation, Japan

**Keywords:** Intestinal failure (IF), Growth impairment (GI), Nutritional intake, Height-for-age z-score (HAZ), Parenteral nutrition (PN)

## Abstract

**Objective:**

Studies investigating growth impairment (GI) in children with intestinal failure (IF) have seldom reported its associated factors. We hypothesized that a deficient nutritional intake would affect GI in children with IF.

**Methods:**

Nineteen patients (2–16 years old) who underwent home-based parenteral nutrition (PN) management at our institution, were divided into patients with or without GI (GI+/GI–). GI was defined as a height-for-age z-score (HAZ) ≤ −2. We performed between-group comparisons of HAZ in the intestinal rehabilitation program (IRP)/nutrition support team (NST) introduction time, PN dependency, residual small intestine length (RSIL), nutritional intake (calorie, protein, lipids, and carbohydrates), caloric intake ratio, and nutrient intake as a percentage of the recommended intake.

**Results:**

Six patients were in the GI+ group. PN dependency was significantly higher in the GI+ group (GI+, 109.8 %; GI < median, 61.7 %, P = 0.036). RSIL did not differ significantly between the groups (50 cm vs. 45 cm, P = 0.879). In the GI+ group, the caloric percentage of lipids was significantly lower (11.9 % vs. 17.5 %, P = 0.002), but that for carbohydrates was significantly higher (76.8 % vs. 66.8 %, P = 0.002), while lipid sufficiency was significantly lower (37.5 % vs. 89.6 %, respectively; P < 0.001). Protein intake in the GI+ group was significantly lower; however, the corresponding sufficiency was > 100 % in both groups (136.5 % vs. 209.2 %, P = 0.005).

**Conclusion:**

For children with IF, a low intake ratio of proteins and lipids relative to energy requirement and required nutrient levels may inhibit adequate growth.

## Introduction

Intestinal failure (IF) is defined as the inability to absorb nutrients and fluids due to short bowel syndrome (SBS) or impaired intestinal motility, requiring parenteral nutrition (PN) for health and growth [1]. In long-term PN management for children with IF, aside from serious complications such as catheter-related bloodstream infections and IF-related liver damage [2], [3], impaired growth and insufficient bone density have also been reported [4], [5], [6], [7]. Accordingly, in addition to avoiding serious complications, assessing growth and aiming for a body composition close to the norm, as well as intervening to promote intestinal adaptation and reduce PN doses, are essential. To better manage children with IF, an intestinal rehabilitation program (IRP) was introduced [8], [9], [10], which has been reported to help avoid complications and improve survival [11], [12]. However, there are no uniform criteria for monitoring indicators in children with IF [13], [14], [15], [16], and related reports on nutritional management are limited. In this study, we assessed the growth, body composition, and nutritional intake in children with IF undergoing PN management at our hospital. We hypothesized that deficiency in nutritional intake is a likely factor affecting GI in children with IF. This study aimed to investigate the association between below-normal growth and nutritional intake.

## Material and methods

### Study design and procedure

This exploratory cross-sectional study analyzed the clinical data of pediatric patients with intestinal failure (IF) who remained dependent on parenteral nutrition (PN) at the time of data collection. Data were obtained from comprehensive assessments conducted at the initiation of home-based PN management by the Intestinal Rehabilitation Program/Nutrition Support Team (IRP/NST) at our institution. Approval was obtained from the institutional ethics committee prior to conducting this study (2023-1-564).

Anonymized clinical data were utilized in this study. This did not involve the use of human-derived samples and was classified as academic research. Consequently, the requirement of informed consent was waived in accordance with the institutional policy. Information regarding the study, including its purpose, was made publicly available on the hospital website to provide patients and their guardians with the opportunity to opt out.

### Patients

Nineteen children aged 2–16 years with IF receiving home-based PN at our institution who underwent their first IRP/NST assessment between January 2013 and May 2023 were included in this study. All patients had been receiving PN at the time of the initial IRP/NST assessment, although the duration of PN prior to the assessment varied among patients. Patients who were successfully weaned from PN were excluded to ensure comparability of nutritional and hepatic parameters. PN discontinuation and advancement to enteral or oral feeding were based on clinical tolerance, stool output, and growth response. Decisions were individualized according to intestinal adaptation, liver function, and overall nutritional status rather than using a fixed institutional protocol.

### Growth index and definition of growth impairment

The growth index was derived from the height-for-age z-score (HAZ), which was calculated using patient height obtained from the hospital’s electronic health records (EHRs) at the time of IRP/NST initiation. We used an automatic computation-enabled Excel file published in 2000 from the Japanese Society of Pediatric Endocrinology website, based on data from the Ministry of Health, Labour and Welfare Report on Infant Physical Growth Survey (age 0–6 years) and the Ministry of Education, Culture, Sports, Science and Technology report on School Health Statistics (age 6–17 years) [17]. According to the World Health Organization (WHO) definition [18], an HAZ of ≤ -2 was used to define GI. In addition to HAZ, the weight-for-age Z-score (WAZ) and BMI-for-age Z-score (BAZ) were calculated to supplement the assessment of growth status. WAZ and BAZ were computed using the same standardized growth references, with WAZ ≤ −2 and BAZ ≤ −2 defined as underweight and thin, respectively.

### Evaluation items

Four evaluation indicators were defined for the group comparison: (i) clinical background factors, (ii) nutritional status, (iii) PN dependence, and (iv) biochemical parameters. These indicators were not identical to the tables but represented pre-specified analytical categories.

(i) *Nutritional intake*

Nutritional intake via three routes—parenteral nutrition (PN), enteral nutrition (EN), and oral nutrition (ON)—at IRP/NST initiation was assessed using EHR data. For each route, daily energy, protein, lipid, and carbohydrate intakes were calculated, and the contribution of each route to the total daily energy intake was determined. For PN and EN, if the same formulation and schedule were used daily, the daily dose was directly recorded. If the formulation or schedule varied by the day of the week, the total weekly dose was divided by seven to determine the average daily intake. For cases without a fixed EN schedule or for ON intake, daily EN volume was calculated from patient- or caregiver-recorded logs over seven days, and ON intake was calculated from three consecutive days of dietary records (including snacks) using the Standard Tables of Food Composition in Japan 2020 (7th revised edition) [19].

To evaluate the adequacy of nutritional intake, energy, protein, and lipid requirements were based on the Dietary Reference Intakes for Japanese, 2020 edition [20], according to sex and age. Energy intake was compared against estimated energy requirements (physical activity level II), protein intake against the recommended intake, and lipid and carbohydrate intake against the midpoint of the recommended percentage of total caloric intake (lipids, 25 %; carbohydrates, 60 %). Each nutrient was expressed as a percentage of the recommended intake.

(ii) *Underlying diseases causing IF*

The underlying diagnosis at the time of IRP/NST initiation was obtained from each patient’s EHR. Based on previous studies, the patients were categorized into the following three groups:

• SBS: midgut volvulus, abdominal wall rupture, trauma, necrotizing enterocolitis, intestinal atresia, or tumor requiring extensive small-bowel resection;
• Intestinal motility disorders: Hirschsprung's disease (HD) or HD-related disorders, chronic idiopathic intestinal pseudo-obstruction, or megacystis microcolon intestinal hypoperistalsis syndrome (MMIHS);
• Mucosal disorders: Crohn's disease, microvillus inclusion disease, malabsorption syndrome, refractory diarrhea, intestinal lymphangiectasia, or protein-losing enteropathy [21], [22], [23].

(iii) *PN dependency and duration*

Resting energy expenditure and PN dependence were calculated using the Schofield equation based on the patient’s body weight at the time of IRP/NST initiation without applying additional stress factors [24], [25]. Parenteral nutrition energy as a percentage of resting energy expenditure was defined as PN dependency [26]. The duration from the start of PN to IRP/NST initiation was defined as the duration of PN.

(iv) *Other clinical variables*

Sex, age at IRP/NST initiation, and residual small intestine length (RSIL) were extracted from patients’ EHRs as background variables.

### Statistical analysis

Continuous variables are expressed as medians with interquartile ranges or ranges, as appropriate, and categorical variables as numbers (percentages). Between-group comparisons were performed using the Mann–Whitney *U* test for continuous variables and Fisher’s exact test for categorical variables. To investigate the factors associated with the presence or absence of growth impairment (GI), we compared GI status with disease classification, PN dependency, residual small intestine length, the proportion of protein, lipids, and carbohydrates relative to total energy intake, and nutrient intake as a percentage of the recommended nutritional requirements. Continuous variables were dichotomized into high and low categories based on the median values, and Fisher’s exact test was used for group comparisons. All statistical analyses were conducted using SPSS Statistics (ver. 29.0, IBM Japan Ltd., Tokyo, Japan).

## Results

### Patient clinical backgrounds and demographic characteristics

Table 1 summarizes the clinical backgrounds and Table 2 presents the demographic characteristics of the 19 patients (11 males and 8 females). The median age at the time of assessment was 7 years. Eight patients were classified into the SBS group, and 11 into the intestinal motility disorder (IMD) group. The median PN duration was 2142 days, median PN dependency was 81.3 %, and median PN glucose intake was 5.5 g/kg/day. Additionally, 14 patients (73.7 %) received an intravenous lipid emulsion and 17 (89.5 %) received oral nutrition. The median RSIL was 50 cm, the median HAZ was −0.93, and 6 patients (31.6 %) had HAZ ≤ −2, indicating GI. To complement the growth assessment, WAZ and BAZ were also evaluated in addition to HAZ. The median WAZ was −1.07, with 7 patients (36.8 %) classified as underweight (WAZ ≤ −2). The median BAZ was −1.1, with 5 patients (21.1 %) classified as thin (BAZ ≤ −2). Among the 6 patients with GI, 5 (26.3 % of the total cohort) were also underweight, whereas 11 patients (57.9 %) had neither GI nor underweight.

**Table 1:** Patient clinical background.

No.	Sex	Age (years)	Disease group	RSIL (cm)	HAZ < -2 (yes/no)	Use of fat emulsion (yes/no)	EN (yes/no)	Oral intake (yes/no)
1	F	2	SBS	35	no	no	yes	yes
2	F	7	IMD	100	no	no	no	yes
3	M	2	SBS	1.5	no	yes	yes	yes
4	M	4	SBS	2	no	yes	no	yes
5	M	5	SBS	70	yes	no	yes	no
6	F	5	IMD	40	no	yes	no	yes
7	M	6	SBS	15	no	yes	yes	yes
8	M	2	IMD	70	yes	yes	yes	yes
9	F	2	SBS	15	yes	yes	yes	yes
10	F	7	IMD	23	yes	yes	yes	yes
11	M	8	IMD	57	no	yes	yes	yes
12	F	8	SBS	6	no	yes	yes	yes
13	M	9	SBS	50	no	yes	no	yes
14	M	9	IMD	unknown	no	no	no	yes
15	M	10	IMD	70	no	yes	yes	yes
16	F	15	IMD	100	no	yes	no	yes
17	M	16	IMD	90	yes	no	yes	yes
18	M	12	IMD	165	no	yes	yes	yes
19	F	12	IMD	unknown	yes	yes	yes	no

Abbreviations: F, female; M, male; SBS, short bowel syndrome; IMD, intestinal motility disorder; RSIL, residual small intestine length; HAZ, height-for-age Z-score; EN, enteral nutrition; ON, oral nutrition.

**Table 2:** Patient characteristics and clinical features (n = 19).

Sex, %	
Male	11 (57.9 %)
Female	8 (42.1 %)
Age, median (IQR), years	7 (4.5–9.5)
Disease, %	
Intestinal malrotation/ midgut volvulus	5 (26.3 %)
Abdominal wall rupture	1 (5.3 %)
Strangulation ileus	1 (5.3 %)
Jejunal atresia	1 (5.3 %)
Hirschsprung's disease [HD]	3 (15.8 %)
HD-related diseases	5 (26.3 %)
MMIHS	3 (15.8 %)
Disease grouping, %	
SBS	8 (42.1 %)
IMD	11 (57.9 %)
Mucosal disorder	0
PN duration (range), days	2142 (118–5134)
PN dependency, median (IQR), %	81.3 (54.2–101.9)
PN glucose intake median (IQR), g/kg/day	5.5 (4.1–8.9)
Using fat emulsion, %	14 (73.7 %)
RSIL, median (IQR), cm (n = 17)	50 (15–70)
HAZ	
Median (IQR)	-0.93 (-2.61 to −0.36)
Category, %	
HAZ ≤ -2	6 (31.4 %)
-2 < HAZ ≤ -1	3 (15.6 %)
-1 < HAZ ≤ 1	9 (47.2 %)
HAZ > 1	1 (5.8 %)
WAZ	
Median (IQR)	-1.07 (-2.36 to −0.65)
Category, %	
WAZ ≤ -2	7 (36.6 %)
-2 < WAZ ≤ -1	3 (15.6 %)
-1 < WAZ ≤ 1	8 (42.0 %)
WAZ > 1	1 (5.8 %)
BAZ	
Median (IQR)	-1.11 (-1.67 to 0.10)
Category, %	
BAZ ≤ -2	4 (21.1 %)
-2 < BAZ ≤ -1	7 (36.8 %)
-1 < BAZ ≤ 1	6 (31.6 %)
BAZ > 1	2 (10.5 %)
Coexisting GI and underweight, %	5 (26.3 %)
No GI or underweight, %	11 (57.9 %)
Total nutritional intake per kg of body weight per day	
Energy, median (IQR), kcal/kg/day	95.0 (68.3–122.1)
Protein, median (IQR), g/kg/day	2.9 (2.6–3.8)
Lipid, median (IQR), g/kg/day	1.9 (1.0–2.1)
Carbohydrate, median (IQR), g/kg/day	15.6 (11.3–21.4)
% energy intake by nutritional intake route	
PN, median (IQR), %	35.6 (23.4–53.3)
EN, median (IQR), %	11.7 (0–19.5)
ON, median (IQR), %	53.0 (41.7–64.2)
Each nutrient as a % of total energy intake, median (IQR), %	
Protein, median (IQR), %	13.6 (12.7–15.6)
Lipid, median (IQR), %	16.1 (14.1–18.4)
Carbohydrate, median (IQR), %	70.1 (66.1–71.4)
Intake as a % of recommendations	
Energy, median (IQR), %	116.0 (92.8–144.7)
Protein, median (IQR), %	168.8 (149.5–225.6)
Lipid, median (IQR), %	62.1 (42.8–96.1)
Carbohydrate, median (IQR), %	127.1 (111.4–165.3)

Abbreviations: IQR, interquartile range; SBS, short bowel syndrome; IMD, intestinal motility disorder; PN, parenteral nutrition; RSIL, residual small intestine length; HAZ, height-for-age Z-score; WAZ, weight-for-age Z-score; BAZ, BMI-for-age Z-score; EN, enteral nutrition; ON, oral nutrition.

The median total energy intake was 95.0 kcal/kg/day, with median protein, lipid, and carbohydrate intakes of 2.9 g/kg/day, 1.9 g/kg/day, and 15.6 g/kg/day, respectively. The median contributions to the total energy intake by route were 35.6 % for PN, 11.7 % for EN, and 53.6 % for ON, indicating that ON was the predominant route of intake. Regarding macronutrient distribution, protein accounted for 13.6 % of total energy intake. Lipids accounted for 16.1 %, which was below the lower end of the recommended target range for fat intake (20 %). In contrast, carbohydrates accounted for 70.1 % of the total energy intake, which exceeded the upper limit of the recommended range (65 %). The median nutrient intake, expressed as a percentage of the recommended intake, was 116.0 % for energy, 168.8 % for protein, and 127.1 % for carbohydrates, all above 100 %. In contrast, the lipid intake was 62.1 % of the recommended level (100 %).

### Comparison of nutritional intake and background indices in the GI+ and GI– groups (Table 3)

In comparisons between the GI+ and GI– groups, no significant differences were observed in sex (male: 50.0 % vs. 46.2 %, P = 1.000), disease distribution (%SBS: 33.3 % vs. 46.2 %, P = 1.000), median age (6 vs. 8 years, P = 0.765), PN duration (1803 vs. 2525 days, P = 0.765), or RSIL (50 vs. 45 cm, P = 0.879). However, PN dependency was significantly higher in the GI+ group (109.8 % vs. 61.7 %, P = 0.036). Both the HAZ (−3.76 vs. −0.44, P < 0.001) and WAZ (−4.42 vs. −0.74, P = 0.009) were significantly lower in the GI+ group.

**Table 3:** Nutritional intake and background factors in the GI+ and GI– group.

	GI+ group (n = 6)	GI– group (n = 13)	*P*-value^a^
Sex, (%)			
Male	3 (50 %)	8 (61.5 %)	
Female	3 (50 %)	5 (38.5 %)	1.000
Age, median (IQR), years	6 (2–12)	8 (5–9)	0.765
Disease group, %			
SBS	2 (33.3 %)	6 (46.2 %)	
IMD	4 (66.7 %)	7 (53.8 %)	1.000
PN duration (IQR), days	1803 (896–2765)	2525 (1293–2774)	0.765
PN dependency, median (IQR), %	109.8 % (69.6–139.1)	61.7 % (45.8–92.6)	0.036
RSIL median (IQR), cm (n = 17)	50 (23–70)	45 (12.5–85)	0.879
HAZ			
median (IQR),	-3.76 (-5.58 to -3.01)	-0.44 (-0.93 to 0.32)	< 0.001
WAZ			
Median (IQR),	-4.42 (-6.68 to -2.08)	-0.74 (-1.45 to -0.55)	0.009
Percentage of energy intake by nutritional intake route			
PN, median (IQR), %	59.3 % (55.2–73.0)	30.0 % (19.9–37.5)	0.002
EN, median (IQR), %	16.3 % (11.0–24.3)	11.2 % (0–17.2)	0.244
ON, median (IQR), %	24.4 % (0–43.4)	58.2 % (52.5–65.7)	0.003
Each nutrient as % of intake energy, median (IQR), %			
Protein, median (IQR), %	11.2 % (9.6–13.1)	14.8 % (13.6–15.7)	0.058
Lipid, median (IQR), %	11.9 % (6.6–14.2)	17.5 % (16.0–21.1)	0.002
Carbohydrate, median (IQR), %	76.8 % (71.1–85.1）	66.8 % (65.2–70.4)	0.002
Intake as a % of recommendations			
Energy, median (IQR), %	96.8 % (64.4–150.7)	118.6 % (97.2–138.7)	0.323
Protein, median (IQR), %	136.5 % (109.3–165.0)	209.2 % (160.0–246.0)	0.005
Lipid, median (IQR), %	37.5 % (27.2–39.5)	89.6 % (62.1–109.2)	< .001
Carbohydrate, median (IQR), %	123.9 % (72.9–213.6)	127.1 % (114.0–153.6)	1.000

Abbreviations: IQR, interquartile range; SBS, short bowel syndrome; IMD, intestinal motility disorder; PN, parenteral nutrition; RSIL, residual small intestine length; HAZ, height-for-age Z-score; WAZ, weight-for-age Z-score; EN, enteral nutrition; ON, oral nutrition.

a Mann-Whitney *U* test.

For the percentage of energy intake by route, PN contributed a significantly greater proportion in the GI+ group (59.3 % vs. 30.0 %, P = 0.002), whereas ON contributed significantly less (24.4 % vs. 58.2 %, P = 0.003). Regarding the macronutrient distribution, the GI+ group had a significantly lower percentage of energy from lipids (11.9 % vs. 17.5 %, P = 0.002) and a significantly higher percentage of energy from carbohydrates (76.8 % vs. 66.8 %, P = 0.002). The protein percentage did not differ to a statistically significant extent (11.2 % vs. 14.8 %, P = 0.058) but tended to be lower in the GI+ group. For nutrient intake as a percentage of the recommended intake, protein intake was significantly lower in the GI+ group (136.5 % vs. 209.2 %, P = 0.005), although both groups exceeded 100 %. Lipid intake was significantly lower in the GI+ group (37.5 % vs. 89.6 %, P < 0.001) and was below 100 % in both groups. The total energy intake tended to be lower in the GI+ group (96.8 % vs. 118.6 %, P = 0.323), whereas carbohydrate intake did not differ to a statistically significant extent (123.9 % vs. 127.1 %, P = 1.000).

### Factors associated with the presence or absence of GI (Table 4)

In Table 4, continuous variables were categorized as high or low using the cohort median as the cutoff value. In the GI+ group, a significantly greater proportion of patients had lower protein intake relative to total energy (5/6, 83.3 % vs. 4/13, 30.8 %; P = 0.010) and lower lipid intake relative to the recommended intake (6/6, 100 % vs. 3/13, 23.1 %; P = 0.003) than in the GI group.

**Table 4:** Association between GI and patient background factors and high/low nutritional intake.

	GI+ group (n = 6)	GI– group (n = 13)	*P-*value^a^
Disease group, %			
SBS	2 (33.3 %)	6 (46.2)	
IMD	4 (66.7 %)	7 (53.8 %)	1.000
PN dependency, %			
high	5 (83.3 %)	6 (46.1 %)	
low	1 (18.7 %)	7 (53.9 %)	0.177
RSIL, % (n = 17)			
high	3 (60 %)	6 (50 %)	
low	2 (40 %)	6 (50 %)	1.000
Each nutrient as a % of intake energy, median (IQR)			
Protein, %			
high	1 (16.7 %)	11 (84.6 %)	
low	5 (83.3 %)	2 (15.4 %)	0.010
Lipid, %			
high	1 (16.7 %)	9 (69.2 %)	
low	5 (83.3 %)	4 (30.8 %)	0.057
Carbohydrate, %			
high	5 (83.3 %)	5 (38.5 %)	
low	1 (16.7 %)	8 (61.5 %)	0.141
Intake as a % of recommendations			
Energy, %			
high	2 (33.3 %)	8 (61.5 %)	
low	4 (66.7 %)	5 (38.5 %)	0.350
Protein, %			
high	1 (16.7 %)	9 (69.2 %)	
low	5 (83.3 %)	4 (30.8 %)	0.057
Lipid, %			
high	0	10 (46.9 %)	
low	6 (100 %)	3 (23.1 %)	0.003
Carbohydrates, %			
high	3 (50 %)	7 (53.8 %)	
low	3 (50 %)	6 (46.2 %)	1.000

Continuous variables were dichotomized into high and low groups based on the median values of the entire study cohort. “High” indicates values equal to or above the median, and “Low” indicates values below the median.

Abbreviations: GI, growth impairment; SBS, short bowel syndrome; IMD, intestinal motility disorder; PN, parenteral nutrition; RSIL, residual small intestine length.

a Fisher’s exact test.

## Discussion

When considering the lifelong prognosis of children with IF, whose survival has improved in recent years [12], ensuring adequate nutrition during childhood is essential for healthy growth. Although several studies have investigated GI in children with IF, few have examined the relationship between nutritional intake and growth outcomes. Therefore, in this study, we aimed to clarify the association between nutritional intake and growth in children with IF.

We found that although PN dependency was significantly higher in the GI+ group, there were no significant differences or detectable associations with RSIL or disease type distribution between the GI+ and GI— groups. Previous studies have reported that patients with IMD have a longer PN duration [27] and higher PN dependency than those with SBS. PN dependency has also been negatively associated with nutritional status [26]. Although it is assumed that a shorter residual small intestine impairs nutrient absorption and increases PN dependence, animal studies have demonstrated that, following small bowel resection, intestinal adaptation occurs through mechanisms such as bowel lengthening and mucosal thickening and proliferation [28]. In addition, in adult patients with SBS, the increased expression of peptide transporter 1 in the colon has been shown to enhance protein absorption [29]. These findings indicate that after intestinal resection, PN dependency is influenced not only by residual bowel length but also by the degree of intestinal adaptation and the intestinal function. In adult SBS, prolonged EN administration has been shown to enhance intestinal absorption of energy, proteins, and lipids, even when combined with oral intake, likely due to sustained stimulation of the intestinal tract [30]. In our study, 17 of the 19 patients consumed oral diets; thus, long-term exclusive EN was not feasible. However, frequent small oral feeding and sustained EN use may promote intestinal adaptation and enhance nutrient absorption.

In terms of nutritional intake, the American Society for Parenteral and Enteral Nutrition (ASPEN) recommends providing approximately 150 % of the estimated energy and protein requirements for patients with SBS given the potential for insufficient intestinal absorption [31]. In our study, although both groups achieved approximately 100 % of the estimated energy requirement, neither group reached 150 %. Regarding protein, the GI-group received 209 % of the estimated requirement, whereas the GI+ group received only 136 %. Furthermore, with respect to macronutrient composition, patients in the GI+ group had a lower percentage of lipids and a higher percentage of carbohydrates in the total energy intake. The appropriate macronutrient distribution has been reported as 20–30 % of the total energy from lipids and 55–65 % from carbohydrates [20]. In our cohort, the median lipid and carbohydrate contributions to total energy intake were 16.1 % and 70.1 %, respectively. These findings indicate an imbalance in macronutrient composition, characterized by insufficient lipid intake and excessive carbohydrate intake. Specifically, while the percentage of lipid intake was approximately 90 % of the recommended amount in the GI– group, it was only approximately 40 % in the GI+ group, indicating a substantial deficiency. This may be attributable to the higher PN dependency in the GI+ group, as PN formulations are predominantly carbohydrate-based. In this cross-sectional study, reduced protein and lipid intake was observed in patients with GI relative to those without GI impairment. This difference was not solely attributable to PN lipid restriction for the prevention of IFALD. In our cohort, most patients received oral or enteral nutrition, and several patients in the GI group had intestinal motility disorders, characterized by poor oral tolerance. Thus, lower protein and lipid intake likely reflects the combined effects of disease-related feeding limitations and PN lipid restriction. Regarding lipid management, approximately 30 % of the patients did not receive intravenous lipid emulsions, primarily due to IFALD-related restrictions or lower PN dependence. In Japan, fish oil-based lipid emulsions and mixed formulations containing fish oil have not been approved for standard clinical use, and only soybean oil-based emulsions are generally available. Our institution is among the few centers that use fish oil-based emulsions in an investigator-initiated clinical trial, mainly for high-risk infants. Consequently, we emphasize early enteral lipid supplementation whenever intestinal adaptation allows minimization of the risk of essential fatty acid deficiency and GI under these regulatory constraints.

Based on the findings of this study, it may be appropriate to use HAZ ≤ –2 as a first-stage screening criterion for GI, to confirm whether HAZ or WAZ has declined, and to monitor nutritional intake to ensure that total energy and protein intake reach approximately 150 % of the estimated requirement and that the lipid-to-energy ratio reaches at least 20 %. In addition, one factor that may hinder self-management in children with IF is the lack of clear guidance regarding the nutrients that may be insufficient and the extent of supplementation required. Establishing quantified nutritional targets may support the development of self-management skills among these patients and help promote healthy growth.

In addition, micronutrient data, including vitamin D, calcium, and trace elements, were not consistently available and, therefore, were not analyzed. We recognize that these factors are crucial for growth, and routine monitoring has since been implemented in clinical practice. Future prospective studies should include detailed analyses of the micronutrient status and enteral nutrition composition. Although we identified significant associations between nutrient intake and growth, several potential confounders, including age, residual bowel length, underlying disease, presence of stoma, and liver function, may have influenced the growth outcomes. The small sample size limits the ability to perform multivariate analyses. Therefore, future multicenter and longitudinal studies are warranted to validate our findings and clarify the mechanisms underlying growth recovery in patients with intestinal failure.

This study had several limitations. First, the cross-sectional design precludes evaluation of longitudinal changes in growth and intestinal adaptation. Second, the sample size was relatively small, and the patients were heterogeneous in terms of age, disease type, residual bowel length, and PN duration, limiting the applicability of a multivariable analysis. Third, data on micronutrients and detailed enteral nutrient composition were incomplete, which may have affected growth evaluation. Fourth, the residual bowel length was determined from surgical records at the time of the last laparotomy and may not represent the actual functional bowel length at the time of the study evaluation. This is particularly relevant for patients with IMD, many of whom have abnormal enteric ganglion development and undergo stoma creation or resection of non-functional segments during the neonatal period, making precise functional length estimation difficult. Despite these limitations, this study provides valuable insights into the nutritional and clinical characteristics associated with GI in pediatric intestinal failure in Japan’s unique regulatory environment for intravenous lipid emulsions. Larger, longitudinal, and multicenter studies are needed to confirm these observations and to guide evidence-based nutritional management strategies.

## Conclusion

For children with intestinal failure, lower proportions of protein and lipid intake relative to the total energy intake and recommended nutrient requirements may be one of the factors associated with impaired growth and body composition.

## CRediT authorship contribution statement

**Mikiko Fuda:** Resources. **Tanaka Chihiro:** Resources. **Yumi Nishikawa:** Resources. **Manami Sasaki:** Resources. **Motoshi Wada:** Supervision, Resources, Investigation. **Miyuki Takeda:** Resources. **Ryoichi Nagatomi:** Supervision, Methodology. **Naoko Inamura:** Writing – original draft. **Hironori Kudo:** Resources, Investigation. **Megumi Nakamura:** Resources, Investigation. **Ryo Ando:** Resources, Investigation. **Tsuyoshi Sakurai:** Resources, Investigation.

## Patient's Guardian's consent

This retrospective study did not involve human biological specimens. Accordingly, the requirement of informed consent was waived in accordance with the institutional ethics policy. Information about the study, including its objectives, was publicly disclosed, providing patients or their guardians with the opportunity to opt out.

## Ethical statement

Ethics approval has been obtained from the Ethics Committee of the institutional ethics committee prior to conducting this study (approval No. 2023-1-564).

## Declaration of Generative AI and AI-assisted technologies in the writing process

During the preparation of this study, the authors used ChatGPT-5 (OpenAI) as a supplementary tool for English language editing. The tool was employed under the supervision of the authors to improve grammar and clarity. After using this tool, the authors thoroughly reviewed and revised the content and the final version of the manuscript was checked by a native English speaker. The authors are fully responsible for the integrity and accuracy of the final manuscript.

## Funding statement

This research did not receive any specific grant from funding agencies in the public, commercial or not-for-profit sectors.

## Conflict of Interest

The authors declare that there are no conflicts of interest relevant to this article.

## Declaration of Competing Interest

The authors declare that they have no known competing financial interests or personal relationships that could have appeared to influence the work reported in this paper.

## Data Availability

The data that support the findings of this study are not publicly available due to privacy and ethical restrictions involving patient information.
